# Un cas de compression médullaire non traumatique atypique

**DOI:** 10.11604/pamj.2015.20.286.6403

**Published:** 2015-03-24

**Authors:** Lova Hasina Rajaonarison Ny Ony Narindra, Willy Ratovondrainy, Anja Holinoro Randriam Arolahy, Pascal Jacques Rajaonarison, Ahmad Ahmad

**Affiliations:** 1 Service d'Imagerie Médicale, Hôpital Universitaire Joseph Ravoahangy Andrianavalona, Ampefiloha, Antananarivo, Madagascar; 2 Service de Neurochirurgie, Hôpital Militaire de Soavinandriana, Antananarivo, Madagascar; 3 Service de Médecine, Hôpital Militaire de Soavinandriana, Antananarivo, Madagascar

**Keywords:** Compression médullaire non traumatique, dorsale, myéloscanner, Nontraumatic medullary compression, dorsal, myelography

## Abstract

Les compressions médullaires non traumatiques de topographie dorsale sont rares. Nous rapportons un cas atypique de compression médullaire dorsale non traumatique chez un jeune homme de 19 ans présentant une paralysie flasque aréflexique avec amyotrophie importante des membres inférieurs, diagnostiquée au myéloscanner et confirmée en per opératoire.

## Introduction

Les compressions médullaires non traumatiques de topographie dorsale sont rares. Ses étiologies sont dominées par les métastases tumorales [1]. Nous rapportons un cas de compression médullaire dorsale non traumatique chez un jeune

## Patient et observation

**Figure 1:**
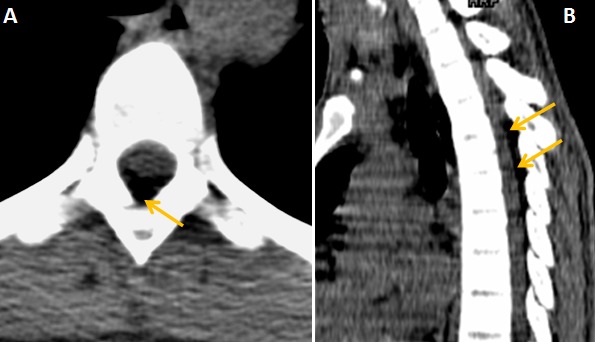
Scanner dorsal sans injection de produit de contraste, coupe axiale (A) et reconstruction sagittale (B). Discrète majoration de la graisse péridurale postérieure en regard de T3 et T4 (flèches)

**Figure 2:**
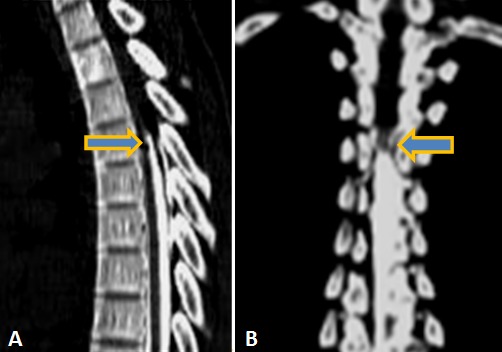
Myéloscanner dorsal, reconstruction sagittale (A) et coronale (B). Arrêt en périphérie du produit de contraste en « dents de peignes » (tête de flèche) à hauteur de T4, faisant évoquer une compression extradurale

**Figure 3:**
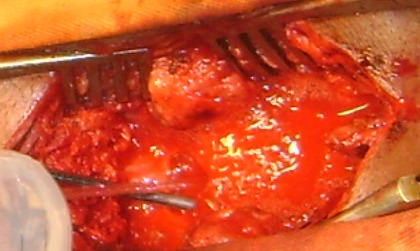
Photographie per opératoire. Tumeur extradurale, friable, très hémorragique

Monsieur Y. O. âgé de 19 ans a été hospitalisé le 03 février 2012 pour une paraplégie évoluant depuis un mois. Il n'avait pas d'antécédent personnel ou familial particulier. A l'interrogatoire, le patient avait quelques mois auparavant une paresthésie résolutive des membres supérieurs et depuis le 26 décembre 2011, une paresthésie des membres inférieurs d’évolution progressive ascendante et s'arrêtant au niveau de la ligne mamelonnaire. L'examen clinique révélait une paralysie flasque aréflexique avec amyotrophie importante des membres inférieurs. On notait la présence de quelques trépidations épileptoïdes du pied gauche. Le reflexe cutanéo-plantaire était indifférent. Il existait des signes d'atteinte de la première racine thoracique (T1) droite de type sensitif associée à une anesthésie en selle et incontinence urinaire, sans notion de fièvre. Le bilan inflammatoire ainsi que le bilan de coagulation étaient normaux. La radiographie du rachis dorsal réalisée en incidences orthogonales était normale. Le scanner cervico-dorsal sans injection de produit de contraste avec reconstruction multiplanaire montrait une discrète majoration de la graisse péridurale postérieure à hauteur des troisième et quatrième vertèbres thoraciques (Figure 1), les éléments osseux étaient normaux. Le myéloscanner réalisé après injection intra-thécale lombaire de produit de contraste montrait un arrêt en périphérie en « dents de peigne » de la colonne opaque à hauteur de la quatrième vertèbre thoracique faisant évoquer une compression médullaire d'origine extradurale (Figure 2). Absence de protrusion discale et les foramen intervertébraux étaient intacts à tous les étages dorsaux. L’étude cytochimique de liquide de ponction lombaire avant l'injection du produit de contraste montrait une dissociation albumino-cytologique sans cellule anormale. Une laminectomie T2, T3, T4 par voie postérieure mettait en évidence une tumeur vascularisée, friable, extradurale, latéralisée à droite, écrasant vers l'avant le fourreau dural et s’étendant de T5 à T1 (Figure 3). L'exérèse tumorale était laborieuse. Le fourreau dural sous-jacent ne présentait plus de battement physiologique habituel. L'examen anatomopathologique de la pièce opératoire montrait une inflammation non spécifique sans signe de malignité. Les suites opératoires immédiates étaient simples; toutefois on notait la persistance du déficit sensitivomoteur des membres inférieurs. Les contrôles évolutifs réalisés au dixième et vingtième jour post-opératoire montraient l’état stationnaire du déficit.

## Discussion

Le syndrome de compression médullaire constitue une urgence diagnostique et thérapeutique [1]. Dans les formes non traumatiques qui sont l'apanage des sujets jeunes de sexe masculin, les étiologies tumorales notamment métastatiques restent majoritaires [1–3] suivies des causes infectieuses surtout dans les pays africains [4]. Les premiers signes cliniques sont dominés par les douleurs rachidiennes qui sont minimes dans le cas rapporté et étaient négligées par notre patient. Puis s'installent les signes déficitaires associant une paresthésie résolutive des membres supérieurs suivie d'une paresthésie des membres inférieurs d’évolution progressive ascendante. Cette paraplégie s'aggrave lentement et s'accompagne d'un trouble sphinctérien. Le liquide de ponction lombaire montre une dissociation albumino-cytologique qui plaide en faveur d'une compression médullaire. La recherche de cellule maligne est souvent négative. La radiographie standard peut situer le siège de la compression en objectivant des lésions osseuses à type de lyse, de condensation ou de tassement vertébral [5]. Les coupes scanographiques en fenêtre osseuse permettent de préciser ces lésions osseuses [5–7]. Chez notre patient, il s'agissait d'une majoration de la graisse péridurale postérieure de l’étage dorsal supérieur épargnant les éléments osseux. La myélographie seule ou couplée au scanner tient une place prépondérante dans la localisation de la compression surtout dans les pays où l'IRM est peu accessible [4] et met en évidence une image d'encoche ou d'arrêt d'aspect variable du produit de contraste selon le siège de l'obstacle. L'arrêt en « dents de peigne » est caractéristique de la compression d'origine extra-médullaire. Toutefois, l'IRM reste l'examen spécifique pour l’étude des lésions intra-canalaires rachidiennes [1, 8]. La chirurgie exérèse a l'avantage d'avoir une confirmation diagnostique [2, 4]. Mais le diagnostic final reste anatomopathologique [4]; il s'agissait, ici, d'une inflammation non spécifique de la graisse épidurale qui reste une affection rare [2].

## Conclusion

La complication inflammatoire d'une lipomatose épidurale peut être source d'une compression médullaire lente avec tableau clinique progressif mais peut être dramatique. Le scanner sans injection permet d'affirmer la majoration de la graisse épidurale et d’éliminer certains diagnostics différentiels avec atteinte osseuse. A défaut d'IRM, le myéloscanner apportera d’éléments utiles au diagnostic.
